# Influence of maxillary molar distalization with clear aligners on three-dimensional direction: molar distal movement, intrusion, distal tip and crown buccal torque

**DOI:** 10.1186/s40510-023-00500-4

**Published:** 2023-12-28

**Authors:** Zeyao Miao, Haijuan Zhang, Yuxuan Yang, Yandong Han, Jing Leng, Shuang Wang

**Affiliations:** 1 Key Laboratory of Shaanxi Province for Craniofacial Precision Medicine Research, College of Stomatology, Xi’an Jiaotong University, Xi’an, Shaanxi China; 2 Department of Orthodontics, College of Stomatology, Xi’an Jiaotong University, Xi’an, Shaanxi China; 3 Clinical Research Center of Shaanxi Province for Dental and Maxillofacial Diseases, College of Stomatology, Xi’an Jiaotong University, Xi’an, Shaanxi China

**Keywords:** Class II, Clear aligners, Molar distalization, Vertical movement, Distal tip, Crown buccal torque

## Abstract

**Background:**

The aim of this study was to evaluate the distal movement, vertical movement, distal tipping and crown buccal torque of maxillary molars after the completion of distalization by comparing the predicted movement with the achieved movement using palatal rugae registration.

**Methods:**

The study included 22 clear aligner patients (7 males and 15 females), and 79 molars were measured. Two digital models were generated before treatment and after molar distalization and were superimposed after selecting the palatal rugae area for registration in GOM inspect suite software 2022 (GOM; Braunschweig, Germany). The predicted and achieved movements of molar distalization, intrusion, distal tip and crown buccal torque were measured and compared.

**Result:**

The achieved distalization (1.25 ± 0.79 mm vs. 2.17 ± 1.03 mm, *P* < 0.001; 1.41 ± 1.00 mm vs. 2.66 ± 1.15 mm, *P* < 0.001), intrusion (0.47 ± 0.41 mm vs. 0.18 ± 0.54 mm, *P* < 0.01; 0.58 ± 0.65 mm vs. 0.10 ± 1.12 mm, *P* < 0.01), distal tip (5.30 ± 4.56° vs. 1.53 ± 2.55°, *P* < 0.001; 4.87 ± 4.50° vs. − 1.95 ± 4.32°, *P* < 0.001) and crown buccal torque (1.95 ± 4.18° vs. − 1.15 ± 4.75°, *P* < 0.001; 0.43 ± 4.39° vs. − 4.27 ± 6.42°, *P* < 0.001) were significantly different from the predicted values in the two groups (first molar, second molar). Significant regression relationships were found between the achieved distal movement and deviational intrusion (*R*^2^ = 0.203, *P* < 0.0001), distal tip (*R*^2^ = 0.133, *P* < 0.001) and crown buccal torque (*R*^2^ = 0.067, *P* < 0.05). There was a significant correlation between the deviational movements of intrusion and the distal tip (*R* = 0.555, *P* < 0.0001).

**Conclusion:**

Approximately 2 mm maxillary molar distalization was achieved in this study. Deviational movement of intrusion, distal tip and crown buccal torque beyond the clear aligner virtual design appeared to a certain degree after distalization. Thus, more attention should be given to molar intrusion and distal tip and crown buccal torque as the designed distalization increases.

## Introduction

For Class II malocclusion patients with maxillary dentoalveolar protrusion or minor skeletal discrepancies, maxillary molar distalization is one of the most common strategies to resolve Class II molar relationships applied in nonextraction treatment [1].

Extraoral traction (i.e., headgear) has been the most common appliance used to achieve maxillary molar distalization since the 1950s [2]. However, patient compliance and cooperation with prescribed intraoral and extraoral devices (i.e., removable functional appliances, intermaxillary elastics, and headgear) are critical for effective distalization [3, 4]. The molar tips distally during distalization with extraoral traction rather than exhibiting bodily movement [5]. Additionally, molar distal tipping, an increase in the buccal rotation, a lower anterior face height and clockwise mandibular rotation have been reported in patients with distal jet and pendulum appliances [5–9]. These side effects increased in the vertical dimension and are disadvantages for Class II patients, in particular hyperdivergent cases [1, 10].

In recent years, an increasing number of adult patients have undergone orthodontic treatment for aesthetic reasons. Clear aligners (CAs) have become a common choice due to their aesthetic and comfort advantages compared with fixed appliances [11]. Initially, CAs were used in simple malocclusion cases, such as slight crowding or minor space closure [12]. With the development of the clear aligner technique, an increasing number of complex cases, such as extractions, open bite and Class II malocclusions, have been treated using this technique [13].

With the presence of attachments on the tooth surface, the predictability of maxillary molar distalization movement can reach 88% in the treatment of Class II malocclusion [14]. Only two groups have investigated the influence of molar distalization with clear aligners on the occlusal vertical dimension. Both evaluated the dentoskeletal vertical dimension with lateral cephalometric radiographs before and after distalization treatment. However, errors in the radiographs and marks could be created by the X-ray operator and measurement expert. Silvia Caruso reported a significant vertical change in molars that contradicted Serena Ravera’s report [15, 16]. Additionally, the linear and angular movements in the buccolingual, mesial–distal and occlusogingival directions designed by orthodontists were ignored in their studies.

The efficiency of tooth movement in clear aligners has been evaluated by the American Board of Orthodontics objective grading system, peer assessment rating systems, cone-beam computed tomography, cephalometric evaluation and superimposition of pretreatment and posttreatment digital models with some superimposition techniques [17–28]. With the development of digital technology, digital models play an important role in evaluating therapy outcomes, particularly clear aligner treatment (CAT) under digital superimposition [29–31]. The palatal rugae area has been reported to be a stable and reproducible structure for superimposition, and palatal rugae registration has been used to evaluate maxillary molar distalization with clear aligners [27, 28].

Therefore, the aim of this study was to evaluate the distal movement, vertical movement, distal tipping and crown buccal torque of maxillary molars after distalization by comparing the predicted movement with the achieved movement using palatal rugae registration in three-dimensional space.

## Methods

### Subjects

This prospective study included 22 participants (7 males and 15 females; mean age: 25.94), and 79 maxillary molars (38 first molars and 41 second molars) were measured using the participants’ digital models. Each participant was treated with sequential distalization [15] in Anglealign (EA Medical Instruments, Shanghai, China) by an experienced orthodontist certified in Invisalign treatment in the orthodontics department. The clear aligner design was completed by the same orthodontist.

The participants met the following criteria: (1) adult patients treated with clear aligners; (2) patients without previous orthodontic experience; (3) maxillary molar distalization was designed; (4) absence or previous extraction of maxillary third molars; (5) standardized treatment protocol and good compliance during the treatment; and (6) high-quality initial digital model with clear palatal rugae structure. This study was approved by the ethics committee (2023-XJKQIEC-003–002).

A power analysis using the efficacy of maxillary molar distalization as the primary outcome was performed on the basis of the results of Simon’s report [32]. Using an alpha of 0.05 and 80% power, a total sample size of 25 molars was needed.

Sequential distalization was used in this study, which means that the aligners are set up to distalize the second molar at first; once the second molars have moved half of the way, then the first molars move back. When the first molars moved half of the way, the second molar arrived at the designed position, then the premolars move and so on [15]. The designed movement distance for each step was 0.2 mm.

To prevent loss of anchorage, microimplants (tapered 1.4 mm; 8 mm long; Ormco, Glendora, Calif) were placed buccally between the roots of the maxillary second premolar and first molar, and Class I elastics (3/16 in., 4.5 oz Ormco Corp., Glendora, CA, USA) were used to reinforce the anchorage. The Class I elastics were worn from the microimplant to the aligners, cut between the lateral incisors and canines. Participants were instructed to wear the aligners for 22 h a day and replace them every 14 days. The participants’ compliance was confirmed, and they were informed that they were part of this study at the aligner delivery appointment.

The mean number of aligners used to achieve maxillary molar distalization was 15.2, and 25 first molars (65.8%) and 20 second molars (48.8%) were designed with attachments.

### Digital setup analysis

The initial models were acquired before the clear aligner treatment, and the final stage models were acquired after maxillary molar distalization. (The specific step which the first molar distalization finished was accessible in the virtual treatment plan, and the intraoral scan was performed once the step completed.) All the actual models were gathered with the iTero Element 2 (Align Technology) intraoral scanner and exported from orthoCAD software (Align Technology) as STL files. The predicted movements of each patient’s tooth were obtained from the tooth movement table provided with the virtual treatment plan.

**Fig. 1:**
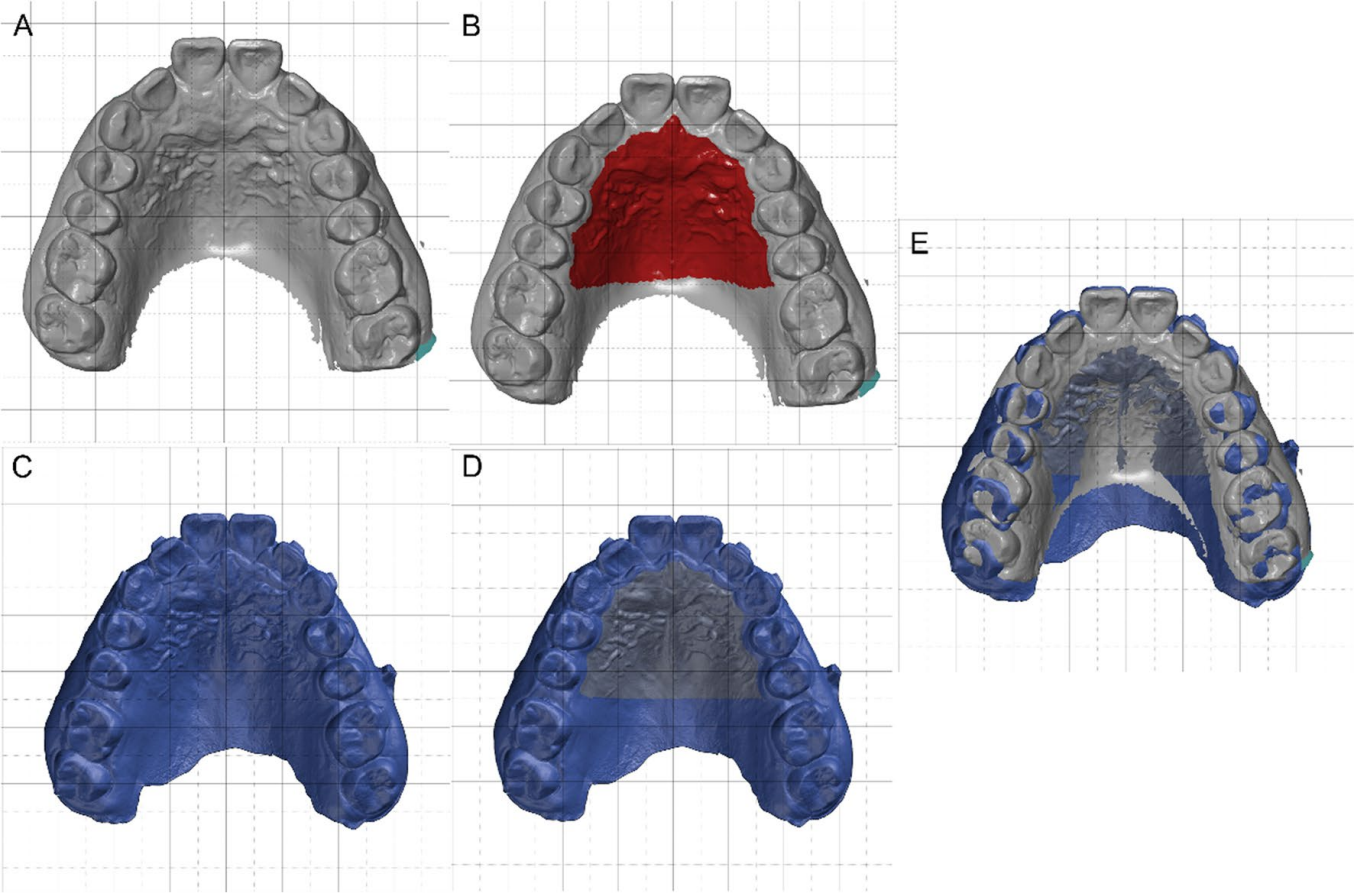
The superimposition of the pretreatment model and the model after distalization. **A** Pretreatment. **B** Selecting the palatal rugae area (pretreatment). **C** Second scan after maxillary molar distalization. **D** Selecting the palatal rugae area (after distalization). **E** Local best fit after selecting the palatal rugae area of the two models

All the actual models were superimposed, inspected and measured after being imported into GOM inspect suite software 2022 (GOM; Braunschweig, Germany). First, prealignment was used to superimpose the initial model and final stage model. Then, the final digital model superimposition was finished with the local best-fit function after selecting the palatal rugae area of the two models (Fig. 1).

**Fig. 2:**
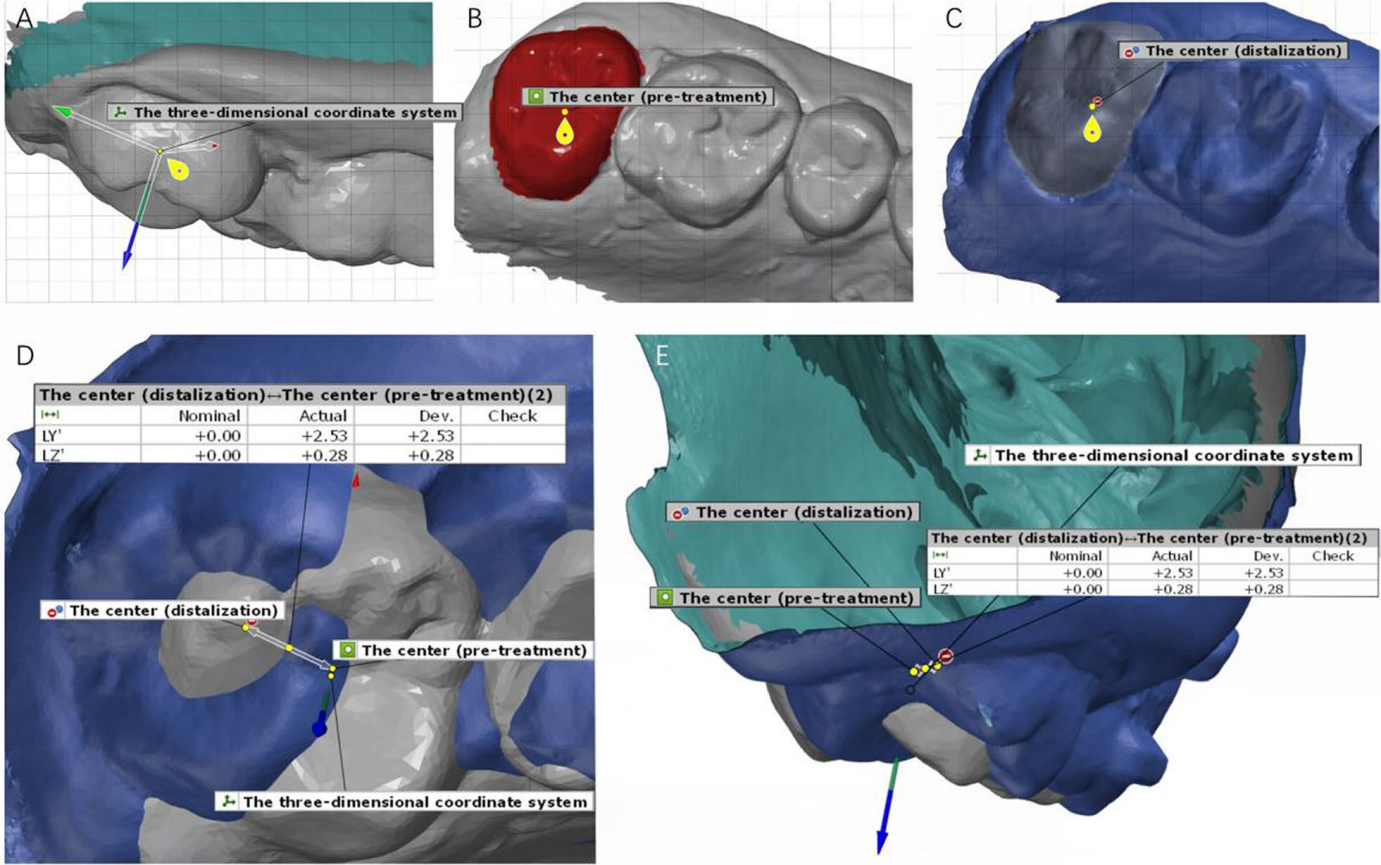
Measurement of maxillary molar linear movement after distalization. **A** The three-dimensional coordinate system was developed according to the morphology of the target teeth. **B** The center of the aimed molar (pretreatment) was constructed by selecting the region of the teeth. **C** The center of the target molar (after distalization) was constructed by selecting the region of the teeth. **D** The distance between the two mass centers was projected to the three-dimensional coordinate system of the target teeth and measured. The mesial–distal movement was obtained after projecting the distance to the Y-axis, i.e., the actual distal movement. **E** The distance between the two mass centers was projected to the three-dimensional coordinate system of the target teeth and measured. The vertical linear movement was obtained after projecting the distance to the Z-axis, i.e., the actual intrusion

After selecting the area of the tooth, the three-dimensional coordinate system was developed according to the morphology of the target teeth, which was the same as that used to obtain the tooth movement table. The buccolingual direction was presented as the X-axis, the mesial–distal direction as the Y-axis, and the occlusogingival direction as the Z-axis (Fig. 2).

**Fig. 3:**
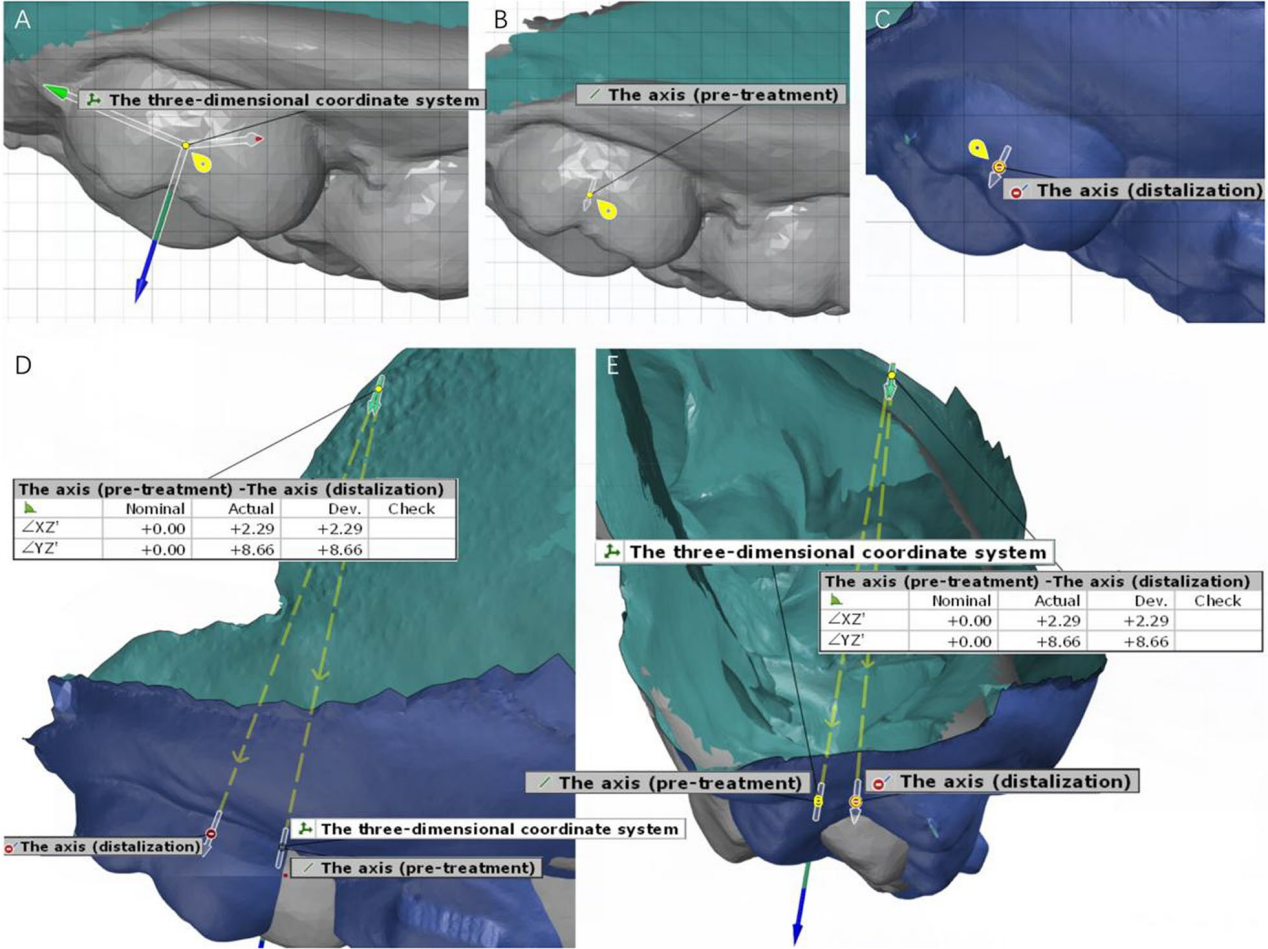
Measurement of maxillary molar angle movement after distalization. **A** The three-dimensional coordinate system was developed according to the morphology of the target teeth. **B** The axis of the target molar (pretreatment) was constructed by selecting the region of the teeth. **C** The axis of the target molar (after distalization) was constructed by selecting the region of the teeth. **D** The angle between the two axes was created and projected to the Y–Z plane in the three-dimensional coordinate system of the target teeth and measured, showing the actual distal tipping. **E** The angle between the two axes was created and projected to the X–Z plane in the three-dimensional coordinate system of the target teeth and measured, showing the actual crown buccal torque

The mass center and the axis of the first and second molars in the three-dimensional direction were constructed over the two models by selecting the region of the target tooth and using the Chebyshev best-fit method. (The mass center is the center of tooth crown mass. The complex molar morphology is simplified to a point and the mass center was used to evaluate the linear movement. The axis of tooth is the geometric axis that runs longitudinally through the tooth and passes through the center of the tooth.) Then, the distance between the two mass centers was projected to the three-dimensional coordinate system of the target teeth and measured. The mesial–distal and vertical linear movements were obtained after projecting the distance to the Y- and Z-axes. The angle (the axis of molar passed the molar mass center and the measured angle was created by extending and translating the two axes in the three-dimensional direction) between the two axes was projected to the Y–Z plane and X–Z plane in the three-dimensional coordinate system of the target teeth and measured, showing molar mesial–distal tipping and buccolingual torque [28] (Figs. 2, 3).

The deviational movement of distal movement, intrusion, distal tip and crown buccal torque was calculated using the following equation: deviational movement = achieved movement – predicted movement. The deviational movement referred to the actual difference between the achieved and predicted movements in the target tooth. Additional movement was achieved compared with the CAT design; the figure was positive, and there was insufficient movement for a negative figure.

The accuracy for achieved distal movement was evaluated using the following equation:

$${\text{percentage of accuracy}}\, = \,{1}00\% {-\!\!-}\left[ {\left( {{\text{predicted}} - {\text{achieved}}} \right)/{\text{predicted}}} \right)\, \times \,{1}00\% ].$$

### Statistical analysis

SPSS software (version 22; IBM Corp., Armonk, NY) was used for the statistical analysis. Means and standard deviations (SDs) were used in the descriptive statistics. The normality assumption of the data was evaluated by the Shapiro‒Wilk test, and the data followed a normal distribution.

The differences in distal movement, intrusion, distal tip and crown buccal torque between the achieved and predicted movements in each group (first molar, second molar) were evaluated with a paired-samples *t* test. The deviational movements of distal movement, intrusion, distal tip and crown buccal torque between teeth with and without attachment in groups (first molar, second molar) were evaluated with an independent-samples t test. Then, stepwise regression was used in the multivariable linear regression model (MLRM) to investigate the relationships between deviational movement (distal movement, intrusion, distal tip and crown buccal torque) and various associated factors (attachments, sex, side of molar, age and SN⋀GoGn).

Linear regression was used to evaluate the relationships between achieved distal movement and deviational intrusion and distal tip and crown buccal torque. Additionally, the pairwise relationships among maxillary molar deviational movements of intrusion, distal tip and crown buccal torque were determined in linear correlation. The level of significance was set at *P* < 0.05.

The reproducibility of measurements was evaluated using the intraclass correlation coefficient (ICC). The same examiner and another examiner remeasured 20% of the models twice after a week to test intrarater and interrater reliability. The intrarater reliability value was 0.949 (*P* < 0.001), which showed excellent agreement, as did the interrater reliability, with a score of 0.953 (*P* < 0.001).

## Results

The descriptive statistics of the sample are shown in Table 1. The achieved, predicted and deviational movements of 79 maxillary molars (38 first molars, 41 second molars) were evaluated for distal movement, intrusion, distal tip and crown buccal torque. The means and standard deviations (SDs) of the achieved, predicted and deviational molar movements (distal movement, intrusion, distal tip and crown buccal torque) are shown in Tables 2 and 3.

**Table 1:** Descriptive statistics of the sample

	Mean	SD
Age	25.9	8.6
SNA (°)	81.4	3.8
SNB (°)	77.8	3.9
ANB (°)	3.6	2.0
SND (°)	75.3	4.1
SN⋀GoGn (°)	27.9	8.4
FMA (°)	24.1	7.5

**Table 2:** Descriptive statistics of the predicted, achieved and deviational molar movements

	Achieved	Predicated	Deviational movement
	Mean ± SD	Mean ± SD	Mean ± SD
Distal movement (mm)	1.34 ± 0.90	2.43 ± 1.11	− 1.10 ± 0.84
Intrusion (mm)	0.53 ± 0.55	0.14 ± 0.89	0.39 ± 0.91
Distal tip (°)	5.08 ± 4.51	− 0.28 ± 3.96	5.36 ± 5.57
Crown buccal torque (°)	1.16 ± 4.33	− 2.77 ± 5.86	3.93 ± 6.05

Significant differences in distal movement (*P* < 0.001, *P* < 0.001), intrusion (*P* < 0.01, *P* < 0.01), distal tip (*P* < 0.001, *P* < 0.001) and crown buccal torque (*P* < 0.001, *P* < 0.001) between the achieved and predicted movements were found in each group (first molar, second molar) (Table 3).

**Table 3:** Differences between the predicted and achieved movements

	Tooth	Achieved	Predicated	*P*
		Mean ± SD	Mean ± SD	
Distal movement	Maxillary first molar (mm)	1.25 ± 0.79	2.17 ± 1.03	0.000
	Maxillary second molar (mm)	1.41 ± 1.00	2.66 ± 1.15	0.000
Intrusion	Maxillary first molar (mm)	0.47 ± 0.41	0.18 ± 0.54	0.008
	Maxillary second molar (mm)	0.58 ± 0.65	0.10 ± 1.12	0.008
Distal tip	Maxillary first molar (°)	5.30 ± 4.56	1.53 ± 2.55	0.000
	Maxillary second molar (°)	4.87 ± 4.50	− 1.95 ± 4.32	0.000
Crown buccal torque	Maxillary first molar (°)	1.95 ± 4.18	− 1.15 ± 4.75	0.000
	Maxillary second molar (°)	0.43 ± 4.39	− 4.27 ± 6.42	0.000

The deviational distal movements between the first molar with and without attachments differed (*P* = 0.011). There were no significant differences between molars with and without attachments in the deviational movement of intrusion (*P* = 0.810, *P* = 0.520) and distal tip (*P* = 0.795, *P* = 0.732) for the first and second maxillary molars. Although the deviational crown buccal torque between the first molar with and without attachments was not significantly different (*P* = 0.827), the difference in the second molar was significant (*P* < 0.05) (Table 4).

**Table 4:** Differences between maxillary molar deviational movements with and without attachments

	Tooth	Attachment	No attachment	*P*
		Mean ± SD	Mean ± SD	
Distal movement	Maxillary first molar (mm)	− 0.70 ± 0.70	− 1.34 ± 0.69	0.011
	Maxillary second molar (mm)	− 1.37 ± 0.97	− 1.14 ± 0.86	0.416
Intrusion	Maxillary first molar (mm)	0.30 ± 0.60	0.25 ± 0.69	0.810
	Maxillary second molar (mm)	0.37 ± 0.87	0.60 ± 1.31	0.520
Distal tip	Maxillary first molar (°)	3.91 ± 4.38	3.49 ± 5.56	0.795
	Maxillary second molar (°)	7.04 ± 5.29	6.63 ± 6.59	0.732
Crown buccal torque	Maxillary first molar (°)	3.23 ± 5.91	2.85 ± 2.59	0.827
	Maxillary second molar (°)	2.18 ± 6.39	7.10 ± 6.56	0.020

When controlling other associated factors in MLRM, the deviational distal movements between the first molar with and without attachments still differed (B = 0.684, *P* = 0.011), and a significant regression relationship was found in the first molar between deviational crown buccal torque and SN⋀GoGn (B = − 0.211, *P* = 0.034) (Table 5). The relationships between the second molar deviational movement and associated factors are shown in Table 6.

**Table 5:** Relationships between deviational movement of maxillary first molar and associated factors in the MLRM

Maxillary first molar	Distal movement (mm)		Intrusion (mm)		Distal tip (°)		Crown buccal torque (°)	
	B (CI)	*P*	B (CI)	*P*	B (CI)	*P*	B (CI)	*P*
*Attachment*								
Y	0.684(0.167,1.201)	0.011	− 0.037(− 0.499,0.425)	0.871	0.626(− 3.035,4.287)	0.730	0.151(− 3.299,3.602)	0.929
N	Reference		Reference		Reference		Reference	
Sex								
Male	0.082(− 0.475,0.639)	0.766	0.086(− 0.412,0.584)	0.726	1.801(− 2.143,5.746)	0.359	− 2.632(− 6.350,1.086)	0.159
Female	Reference		Reference		Reference		Reference	
*Side of molar*								
Left	− 0.373(− 0.846, 0.101)	0.119	0.021(− 0.402,0.445)	0.919	0.527(− 2.827,3.881)	0.751	− 1.108(− 4.269,2.054)	0.480
Right	Reference		Reference		Reference		Reference	
Age	− 0.004(− 0.036,0.028)	0.789	− 0.022(− 0.050,0.007)	0.133	0.069(− 0.159,0.296)	0.542	− 0.081(− 0.295,0.133)	0.448
SN⋀GoGn	− 0.003(− 0.032,0.026)	0.842	0.010(− 0.016,0.036)	0.434	− 0.055(− 0.260,0.150)	0.588	− 0.211(− 0.404,-0.017)	0.034

**Table 6:** Relationships between deviational movement of maxillary second molar and associated factors in the MLRM

Maxillary second molar	Distal movement (mm)		Intrusion (mm)		Distal tip (°)		Crown buccal torque (°)	
	B (CI)	*P*	B (CI)	*P*	B (CI)	*P*	B (CI)	*P*
*Attachment*								
Y	− 0.306(− 0.923,0.311)	0.320	− 0.116(− 0.895,0.664)	0.765	− 0.223(− 4.199,3.752)	0.910	− 4.348(− 8.885,0.189)	0.060
N	Reference		Reference		Reference		Reference	
Sex								
Male	− 0.055(− 0.735,0.625)	0.871	0.663(− 0.196,1.523)	0.126	0.955(− 3.429,5.339)	0.661	− 0.173(− 5.176,4.831)	0.945
Female	Reference		Reference		Reference		Reference	
*Side of molar*								
Left	− 0.048(− 0.026,0.530)	0.868	0.170(− 0.560,0.900)	0.639	1.654(− 2.071,5.380)	0.374	0.846(− 3.406,5.098)	0.689
Right	Reference		Reference		Reference		Reference	
Age	− 0.028(− 0.072,0.115)	0.194	0.014(− 0.041,0.069)	0.597	0.280(− 0.001,0.561)	0.051	0.027(− 0.293,0.347)	0.867
SN⋀GoGn	− 0.011(− 0.046,0.024)	0.525	− 0.011(− 0.055,0.034)	0.626	− 0.137(− 0.363,0.090)	0.229	− 0.227(− 0.486,0.032)	0.083

**Fig. 4:**
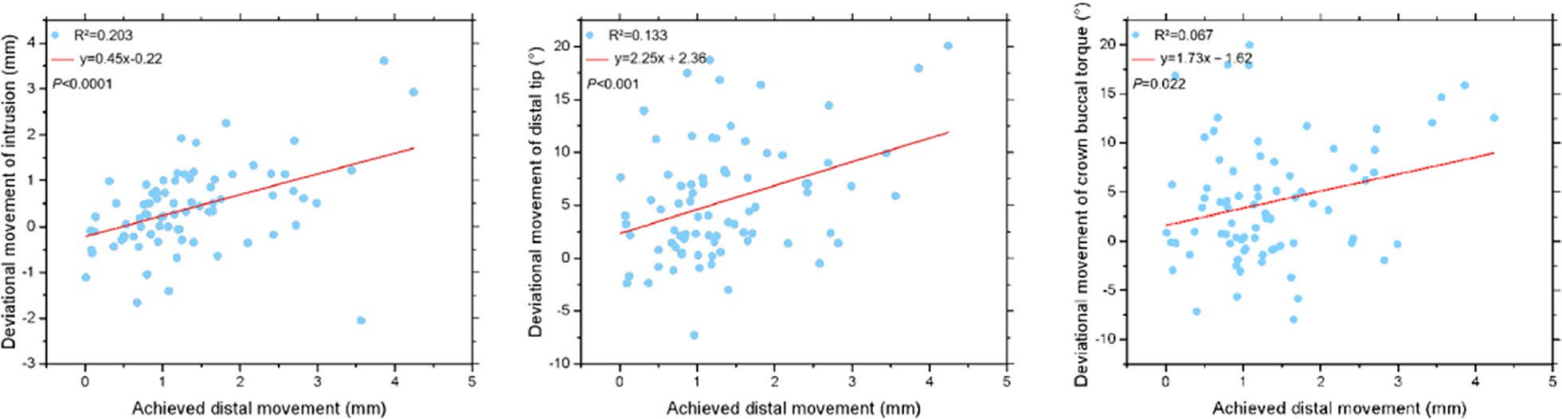
The relationships between achieved distal movement and deviational movements of intrusion, distal tip and crown buccal torque

**Fig. 5:**
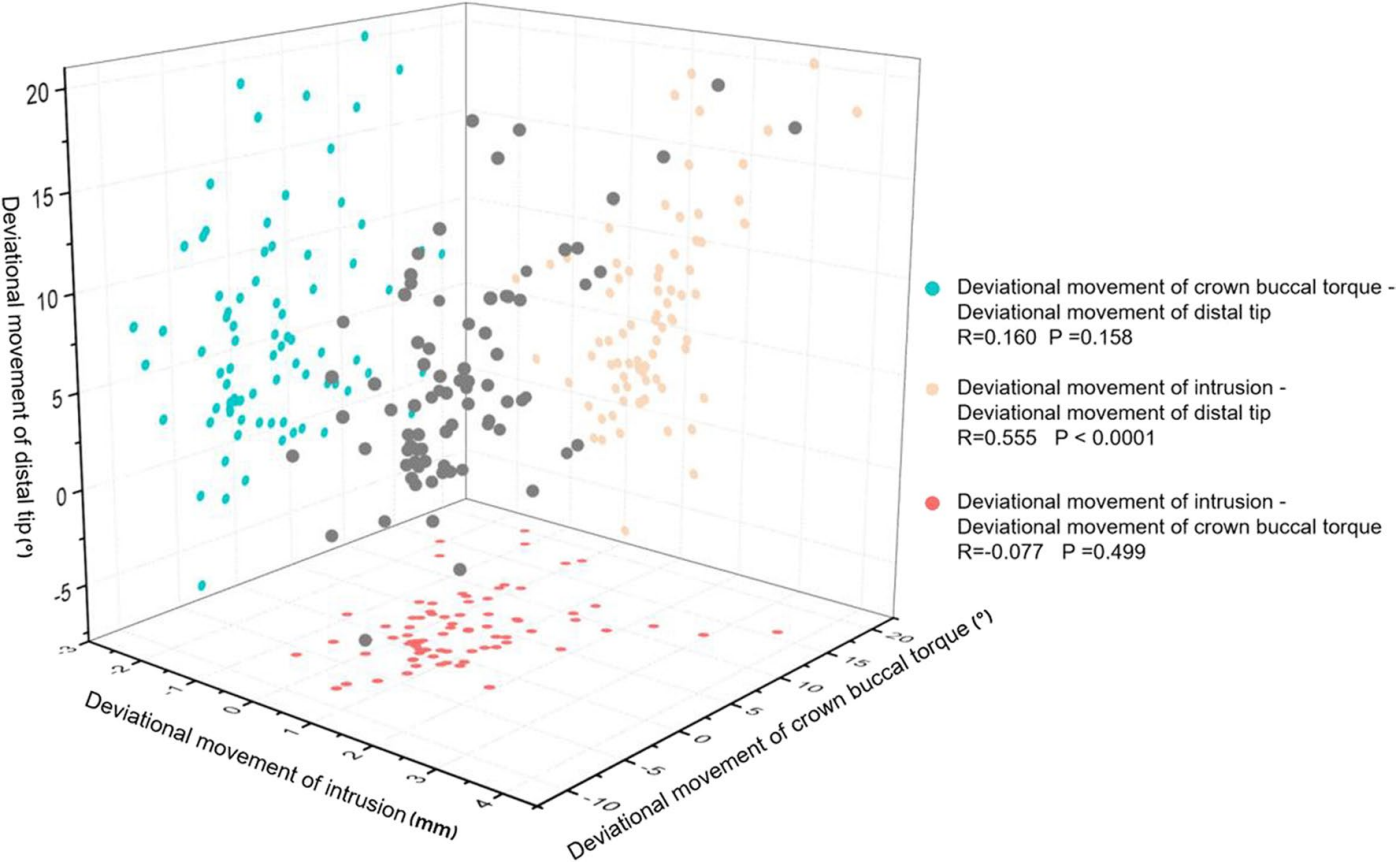
The pairwise relationships among maxillary molar deviational movements of intrusion, distal tip and crown buccal torque

Significant regression relationships were found between achieved distal movement and deviational intrusion (*R*^2^ = 0.203, *P* < 0.0001), distal tip (*R*^2^ = 0.133, *P* < 0.001) and crown buccal torque (*R*^2^ = 0.067, *P* < 0.05) (Fig. 4). There was a significant correlation between the deviational movements of intrusion and the distal tip (*R* = 0.555, *P* < 0.0001) (Fig. 5).

## Discussion

Maxillary molar distalization is a common nonextraction strategy for treating Class II malocclusion patients with maxillary dentoalveolar protrusion or minor skeletal discrepancies, which could improve the molar relationship and achieve a certain retrusion of anterior teeth [1]. Bodily movement is the ideal form of maxillary molar distalization; however, distal tip and buccal torque appear after molar distalization completion when using traditional appliances [6–9]. Additionally, increased buccal rotation was reported in an in vitro study [5]. The degree of distalization achieved with a clear aligner was approximately 2 mm in previous studies [15, 28], and molar distal tip [15] and vertical movement [16] were observed after comparing pretreatment and posttreatment lateral cephalometric radiographs. To the authors’ knowledge, this is the first study to analyze the distal movement, vertical movement, distal tipping and crown buccal torque of maxillary molars after distalization in three-dimensional space.

This study revealed that the overall accuracy of maxillary molar distal movement achieved by a clear aligner amounted to 55.9%, and the achieved distalization was less than the predicted distalization. Simon et al. [32] reported a high accuracy (87%) of maxillary molar distalization, and Saif BS et al. [28] reported an accuracy of 73.8%. More accurate distalization might be achieved by applying proper staging in the virtual treatment plan and designing attachments for the target molar [32]. The material used to produce aligners might also influence the accuracy of tooth movement. Biomechanically, the distalization process is achieved by increasing the aligner volume between the target molar and anchor premolar and placing a mesial–distal force on the mesial adjacency of the target molar. The premolar and anterior teeth are regarded as the anchorage part [33]. Although the distance between the target molar and premolar is the same as the design in the virtual treatment plan after distalization, mesial movement of the premolar, flaring of the incisor [28] and decreasing target molar distalization appear, assuming that the anchorage is insufficient. Consequently, the achievement and accuracy of maxillary molar distalization decrease.

In a previous study, molars moved 2–3 mm vertically, and the mean distal tip was 1.3° [16]. In contrast, Ravera S et al. [15] reported no significant vertical and mesiodistal tipping movements in molar distalization. Here, the achieved distal tip, intrusion and crown buccal torque were significantly higher than the design after distalization. A mean intrusion of 0.39 mm, distal tipping of 5.36° and crown buccal torque of 3.93° were performed in the deviational movement, which showed a certain intrusion, tipping and crown buccal torque beyond the aligner design. The bite block effect caused by the thickness of the aligners and the frequent usage of aligner chews might influence the vertical movement of maxillary molars [34]. Similarly, after distalization, the wedge effect and decreased distance between the molar and hinge movement axis (temporomandibular joint, TMJ) might increase the molar bite force and intrusion movement. The force in molar distalization is inflicted on the crown, which is in the occlusal direction compared with the target molar center of resistance. A certain mesiodistal tipping movement appears due to the deficiency of moments used to control root movement [35]. The increased crown buccal torque during the distalization process can be explained by the buccal alveolar bone, which is thinner than that of the palate, leaving molars more vulnerable to the buccal tip. When controlling other associated factors, the additional crown buccal torque would increase as the SN⋀GoGn decreases in the first molar. Higher alveolar bone density in low-angle patients and poorer aligners surface around the first molar may reduce buccal control. The preventive overcorrection of vertical extrusion, mesial tip and lingual torque might be the solution for this deviational tendency. However, the change in lower anterior face height after molar intrusion and distal tipping was not investigated.

Many auxiliary elements, such as attachments, composite buttons and combinations of microimplants and elastics, have been developed and applied in the clinic to improve the accuracy of clear aligner tooth movement [36]. Ravera et al. [15] reported that the use of attachments improved the movement of the first molar distalization but did not influence the second molar. Additionally, no significant differences were found in another study that evaluated distalization movement between molars (first molar and second molar) with and without attachments [28]. Here, the deviational movements of distal movement, intrusion, distal tip and crown buccal torque were identified and used to compare the effect of attachments. Attachments did not influence the degree of deviational movement (second molar distal movement, intrusion, distal tip and first molar crown buccal torque), indicating that the application of an attachment could not improve second molar distalization. Additionally, with the usage of attachments, the additional intrusion, distal tip and first molar crown buccal torque beyond the virtual design were not reduced. It is assumed that an aligner can fit on the tooth surface better with the usage of attachments. The first molar has less surface in the mesial–distal direction, and the use of attachments increased the distal enclasp force and distal movement accuracy. However, molars have a larger surface area and undercut area in other directions; thus, no significant increase in enclasp force occurred with the use of attachments. When controlling the associated factors (sex, side of molar, age and SN⋀GoGn), the effect that attachments decreased the second molar additional crown buccal torque disappeared. Thus, the usage of attachments alone may not enhance the second molar crown buccal control.

This study revealed that with the increase in achieved maxillary molar distal movement, there was a certain rise in the vertical intrusion, distal tip and crown buccal torque beyond the clear aligner design. Additionally, a significant linear correlation relationship was found between the deviational movements of intrusion and the distal tip. At every stage, 0.2 mm maxillary molar distal movement was designed, and some intrusion, distal tip and crown buccal torque occurred, with molar distal movement achieved due to the bite block effect and deficiency of controlling root movement moments. The deviational movement accumulated stage by stage throughout the aligner treatment and distalization was achieved. We should pay more attention to molar deviational intrusion and the distal tip simultaneously when more distalization is designed.

### Limitations

Patient cooperation, such as the interval between follow-up visits, the time spent wearing appliances and the frequency of using aligner chews, might influence the effect of removable appliances. Additionally, tooth movement might be affected by the patients’ treatment course and alveolar bone thickness. None of the above factors were investigated because of the limited sample size and research method. Although the first and second molars were analyzed separately while the effect of attachments was evaluated and the MLRM was used to control associated factors, the lack of independence between different molars of the same person still existed.

This study focused on Class I and slightly skeletal Class II malocclusion patients, and most samples were average angle patients. Thus, the conclusions of this study were not appropriate for severe skeletal Class II malocclusion patients. The movement tendency of maxillary molar distalization in severe high-angle and low-angle patients might be different from this study.

The final scan digital models were gathered as soon as molar distalization was achieved. Posterior tooth anchorage loss and molar mesial tips might appear with the retrusion of anterior teeth, which would influence the linear and angular movements of maxillary molars. Moreover, miniscrew anchorage to Class I intramaxillary elastics was used after distalization to maintain anchorage during subsequent retraction of the remaining teeth.

Two patients underwent single molar distalization (2 first molars, 1 second molar), and the others underwent dual molar distalization. Although there might be a significant difference between the two groups, the usage of miniscrew anchorage to Class I intramaxillary elastics decreased the variation.

Maxillary molar distalization was followed by increased lower anterior face height, molar distal tip and clockwise mandibular rotation in patients with distal jet and pendulum appliances, which were disadvantages for Class II patients and hyperdivergent cases [6–9]. Although a certain molar intrusion and distal tip were found in clear aligner patients, further study with cone-beam computed tomography (CBCT) after treatment is required to evaluate the skeletal vertical dimension.

## Conclusion

1. In this study, approximately 2 mm maxillary molar distalization was achieved, and a certain degree of deviational movement of intrusion, distal tip and crown buccal torque that extended beyond the clear aligner virtual design appeared after distalization.
2. Attachments did not substantially improve the differences in the second molar distalization, intrusion, distal tip and crown buccal torque between the achieved and predicted movements. However, attachment use improved first molar distal movement. Overcorrection might be a further research interest as a solution.
3. A certain increase in deviational intrusion, distal tip and crown buccal torque occurred with the achievement of maxillary molar distalization, and a synchronously increasing tendency of deviational intrusion and distal tip was observed. Thus, more attention should be given to molar intrusion and distal tip and crown buccal torque with increasing designed distalization.

## Data Availability

The datasets used and/or analyzed during the current study are available from the corresponding author on reasonable request.
